# Methyl and Total Mercury in Different Media and Associated Fluxes in a Watershed Forest, Southwest China

**DOI:** 10.3390/ijerph15122618

**Published:** 2018-11-22

**Authors:** Hongxia Du, Ming Ma, Tao Sun, Siwei An, Yasuo Igarashi, Dingyong Wang

**Affiliations:** 1 College of Resources and Environment, Southwest University, Chongqing 400715, China; duhx@swu.edu.cn (H.D.); maming@swu.edu.cn (M.M.); suntao81589@126.com (T.S.); asw5713176@126.com (S.A.); aigara@mail.ecc.u-tokyo.ac.jp (Y.I.); 2 Chongqing Key Laboratory of Bio-Resource for Bioenergy, Southwest University, Chongqing 400715, China

**Keywords:** THg, MeHg, watershed forest, deposition, transport

## Abstract

Mercury (Hg) deposition in the forest ecosystem is a significant source of input for methyl Hg (MeHg) and total Hg (THg) to the subtropical forest field and downstream aquatic systems. Wet deposition, litterfall, runoff, and fluxes with forest soil percolate of MeHg and THg were sampled for two years in a watershed forest of southwest China. Results showed that the depositions of THg and MeHg through litterfall and throughfall were 86 µg m^−2^ yr^−1^ and 0.8 µg m^−2^ yr^−1^ respectively, with litterfall acting as a predominant route for the input of both THg and MeHg. The estimated fluxes of THg and MeHg in the throughfall and litterfall were 3 and 4 times greater than those in the precipitation. Methylmercury in the decomposed litter migrates during its erosion by surface runoff and the concentrations of MeHg were quite consistent with that in the surface runoff. Methylmercury mainly accumulated in the lower layer of the litter and upper layer of the soil (*O_i_*), and its transfer through the soil cross-section was delayed. THg retention was not consistent with MeHg, probably with lower soil layers (*O_e_* and *O_a_*) storing and enriching THg in the forest ecosystem. The forest floor of the lower soil is an effective sink for THg but not for MeHg. Methylmercury accumulated in decomposing litter and upper soil layer might transfer with soil percolate, possessing potential ecological risks for residents living around the downstream aquatic systems.

## 1. Introduction

Mercury (Hg) is a worldwide environmental contaminant that leads to the contamination of forest, soil, water, plants, snow, and others [1]. It is well accepted that elemental Hg (Hg^0^) can travel long distances in the atmosphere, impacting aquatic systems in remote regions through deposition [2,3,4,5,6]. Consequently, atmospheric deposition is regarded as the principal source of Hg input for remote pristine regions with rare anthropogenic Hg sources [7]. As a general rule, dry deposition, estimated as the sum of litterfall and throughfall minus open-field wet deposition, is more dominant than wet deposition of Hg [8]. Recent research shows that dry deposition is more important than wet deposition in North America over vegetated surfaces [9]. Wet deposition of Hg in the forest is different with the water surfaces and non-forested areas, being largely dependent on the forest types. Mercury cycling in the forest ecosystem received more attention in the past years because the forest field plays crucial roles in the terrestrial cycling of total Hg (THg) and methylmercury (MeHg), acting as a link between the atmospheric and aquatic ecosystems [10,11]. Atmospheric Hg deposition is promoted in the forest system comparing with other ecosystems due to the large surface areas associated with canopy foliage. Moreover, the effective surface areas differ among different types of vegetation, leading to different deposition rates. The total atmospheric Hg deposition can be calculated by the fluxes of throughfall and litterfall [12]. THg levels in the throughfall under boreal forest canopy increase in northern American [13]. The evergreen broadleaf forest canopies have greater surface leaf areas and more surface roughness than the northern American forest, which contributes to slower air flow rate and elevated Hg input fluxes [14]. Therefore, it is hypothesized that more Hg from canopies possibly enters into the forest floor due to the dense canopies of the subtropical forest [14].

Simian Mountain belongs to a typical subtropical forest system located in Chongqing, southwest China. Its subtropical evergreen broadleaf forest is by far the world’s largest and best- preserved forest near 28 degrees north latitude. Although strict controls on industrial emissions have resulted in a significant decrease in Hg levels and acid rain deposition in recent years, those changes may have a lagged impact over time. Besides the natural sources of Hg pollution, anthropogenic sources of Mt. Simian have considerably elevated because of artisanal gold-mining, fossil fuel combustion, as well as pharmaceutical, industrial and chemical applications, especially in booming China over the past 30 years [5,15,16,17,18]. Subtropical forests are characterized by higher forest coverage and dominant perennial evergreen trees. The forest coverage of the researched forest reaches 95.8%, among which 98% is evergreen broadleaf forest, which makes it possess larger surface area to absorb more Hg [19]. Meanwhile, the sub-alpine water conservation forest is the most important drinking water protection site in China. Deposited Hg in the water conservation forest is redistributed by the forest canopies, litterfall and forest soils through infiltration and accumulation into the surface and subsurface runoff, thus providing essential water source for downstream aquatic ecosystems and local residents, especially among those in extreme poverty and remote areas in southwest China. Therefore, it is significant to research on Hg fate in a representative subtropical watershed forest, which is useful for evaluating the risk of Hg for residents living around the downstream aquatic systems.

Scientists have conducted extensive research in the past years in order to interpret the fate and transformations of Hg in the litterfall, throughfall, runoff and soils of watershed forests [20,21,22]. They found potential terrestrial sources of THg and MeHg in the runoff and the processes controlling the load of Hg from soil to the runoff, which indicated that the upland Hg output might possess certain ecological risk [14,23,24]. Our previous research also found that the forest runoff increased the output of Hg from the catchments of Mt. Simian, especially in summer after heavy rains [25]. However, little attention has been paid to the long-term studies on transformation and accumulation of Hg in different organic layers of the soils, and the final fate of Hg in the soil profiles. Therefore, there is a critical need to understand the fate and behavior of Hg in a watershed forest stand and evaluate the ecological risk of Hg to the local residents. Therefore, the objective of this study was to investigate the contribution of elevated atmospheric Hg deposition in a sub-alpine water conservation forest, and the potential Hg output fluxes to downstream aquatic ecosystems where Hg could accumulate to toxic levels in fish and other aquatic animals. On the other hand, we also aimed to investigate whether the differences in the behavior of MeHg and THg among different layers of the forest floor support the hypothesis that Hg, especially the MeHg, may be much easier to transport in the forest floor due to the dense canopies under the warm and watery subtropical climate and the regional elevated atmospheric Hg emission.

## 2. Materials and Methods

### 2.1. Site Description

**Figure 1:**
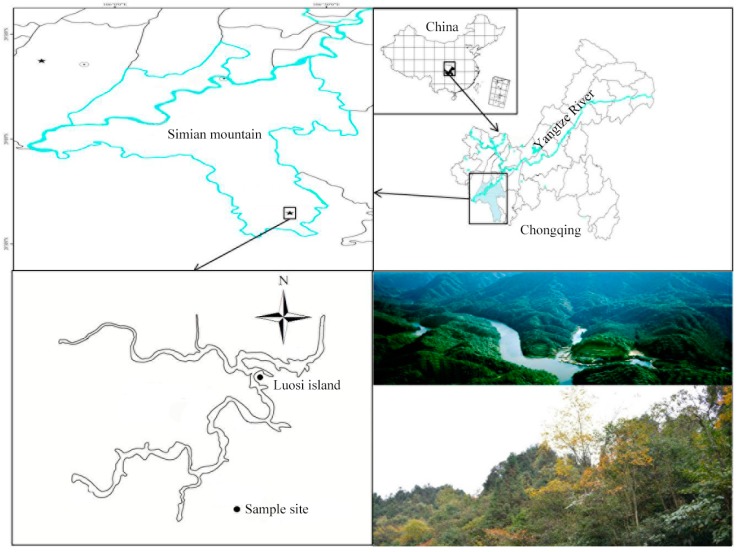
Schematic diagram of the study site, Mt. Simian in Chongqing, China.

Chongqing is located in the southwest of China’s inland and the upstream of Yangtze River. Mt. Simian national nature reserve (106°22′–106°25′ E, 28°35′–28°39′ N) is situated approximately 200 km away from urban Chongqing. The climate of Mt. Simian is predominantly subtropical humid monsoon, with a well-defined annual rain season from June to September. Annual average temperature and rainfall is 13.7 °C and 1127 mm respectively. The evergreen broadleaf forest is one of the most representative and well-preserved vegetation types in Mt. Simian. More importantly, it is regarded as the sole extant, largest forest in the same latitude of the earth. Therefore, a secluded evergreen broadleaf forest stand without anthropogenic sewage was selected as our sample site (Figure 1).

### 2.2. Sampling Method of Throughfall, Runoff and Soil Percolate 

The precipitations were collected by automatic precipitation samplers (APS-3A, Changsha Xianglan Scientific Instruments Company, Changsha, China) settled on an open forest field of the study site. The instrumental standards and the setting parameters of APS-3A were consistent with our previous research [26].

Three permanent observation plots (20 × 20 m^2^) were selected to collect throughfall by self-made collectors, which consisted of acid-washed borosilicate glass bottle, protected by polyvinyl chloride covering systems, and wide-mouth Teflon funnel (20 cm in diameter). The collectors were sealed by polyethylene after each precipitation event, avoiding Hg dry deposition from falling into the sampling bottles. Subsequently, the sampled rain water at the three plots was mixed. The volume-weighted mean concentrations (VWM) were used to describe monthly THg and MeHg concentrations based on Equation (1).

(1) $$\mathrm{VWM}=\frac{X_{1}\times V_{1}+X_{2}\times V_{2}+\cdots +X_{t}\times V_{t}}{V_{1}+V_{2}+\cdots +V_{t}}.$$

where *X_t_* is the concentration of Hg in each precipitation event (ng L^−1^), *V_t_* is the volume of each rainfall (mm).

Hg fluxes were obtained by multiplying Hg concentrations by precipitation volume. Wet deposition fluxes of THg and MeHg were calculated based on Equation (2).

(2) $$F_{w}=\frac{1}{1000}\sum_{i=1}^{i=n}\left(C_{R}^{i}P^{i}\right)$$

where *F_w_* means the annual THg or MeHg wet deposition fluxes (mg m^−2^ yr^−1^), *C_i_* means the VWM (ng L^−1^) of each rain sample, and *P_i_* (mm) means the amount of precipitation or throughfall.

Five random sites for runoff were selected at the study area. In each site, the runoff at the interface of litterfall and soil were collected by five stainless steel water collection plates (20 × 20 cm), and then transferred into borosilicate glass bottles (1 L) by Teflon pipes. During the sampling period, the runoff samples were collected every two weeks. 

Forest-soil percolate was sampled below soil horizon of the forest floor with 9 (15 × 30 cm) lysimeters which were made from pure, non-leaching food grade stainless steel. Forest-floor percolate solutions were sampled at 3 random locations near the throughfall collectors within the study plot, with three lysimeters at each location. At each of the three plots, the plate lysimeters were installed, namely three replicates underneath the *O_i_*, *O_a_* and *O_e_* layers of the forest floor respectively. Prior to their installation in the field, the lysimeters were percolated with standard soil solutions of known Hg concentrations (Table 1). In order to make sure there were no memory effects, the lysimeters were installed in the soil more than three months before the beginning of the forest-floor percolate collections. Since the forest-soil percolate and element fluxes in the organic layer were limited, the water balance fluxes were adjusted by the annual budget of Cl^−^ in the forest floor by the annual sum of weekly measured Cl^−^ fluxes with throughfall [27], and obtained as mean values from five throughfall samplers at each of the three subplots. 

### 2.3. Sampling Method of Litterfall 

Litter traps (1 m × 1 m) with the base bottom covered by perforated plastic sheets were randomly arranged in each forest plots (2 m × 2 m/plot, five plots) in late October 2013 when was the largest amount of litterfall period of the studied subtropical forest. The litter bag technique was adopted to calculate the decomposition rates. The litters obtained by the traps were put in the acid-cleaned litter bags (10 cm × 10 cm), with 10 g litters per bag. A total of 60 L bags were produced and randomly placed on the plantation floors of a larger plot of 20 m × 20 m in October 2013. Mesh size of 1 mm was used, because free entry of small soil animals was allowed and microbial activities were kept. Finally, a total of 48 L bags containing decomposing litters were obtained from the forest floor for two years (724 days), with 2 bags monthly.

### 2.4. Sample Analysis, Quality Control and Statistical Analysis

In this study, all containers and tubes were rinsed thoroughly with ultra-pure water (Milli-Q integral 3, Millipore, France) after being soaked in dilute 10% (*ν*/*ν*) HNO_3_ for 24 h. Borosilicate containers, such as bottles and large-mouthed jars, were broiled at 500 °C for 40 minutes in a muffle furnace after soaking and rinsing, and then parceled by three PE preservation bags when they were cooling in a sealed ultra-pure testbed. Lastly, they were housed in a box to keep the temperatures of bottles and jars from getting too high. Especially, PE gloves were obliged to equip during all sampling.

THg and MeHg concentrations in the water samples were measured by the EPA Method 1631 and 1630 respectively [28,29]. THg concentrations in the leaf tissue were measured by cold vapor atomic absorption spectrophotometry (CVAAS) after a series of treatments, including acid digestion, oxidation, purge and trap [26]. THg concentrations in the soil were measured by a DMA-80 (Milestone, Sorisole, Italy) direct Hg analyzer, with detection limit of 0.005 ng. Standard reference material for soil component analysis, soils in Sichuan Basin (GBW07428/GSS-14, see www.gbw365.com) was used to make standard and calibration during the measurement of soil THg. The concentrations of MeHg in the soil and leaf tissue were measured by cold vapor atomic fluorescence spectrophotometry (CVAFS) after a series of pre-treatments, such as digestion, extraction and back-extraction in accordance with EPA Method 1630. The mechanisms for measuring MeHg and EthylHg are based on the different separation coefficients of different species of Hg driven by carrier gas (high purity argon) in Hg analyzer.

The matrix spikes recoveries were 89% to 113% for THg, and 91% to 116% for MeHg respectively. The average relative standard deviation for repeated analyses were 5.2% for THg and 5.4% for MeHg. The precisions of duplicate samples for THg in water, soil and leaf tissues were 5%, 9%, and 4% respectively, while for MeHg, 4%, 7% and 8%. The method blank for THg and MeHg were 0.05–0.1 ng L^−1^ and 0.03–0.01 ng L^−1^ respectively, which were both far lower than the sample concentrations. Student’s two-tailed *t*-test is used for statistical analysis to determine whether two sets of data are significantly different from each other, with *p* < 0.05 being considered as statistically significant.

## 3. Results

### 3.1. Influence of the Forest Canopy on Hg Deposition through Throughfall and Litterfall

**Figure 2:**
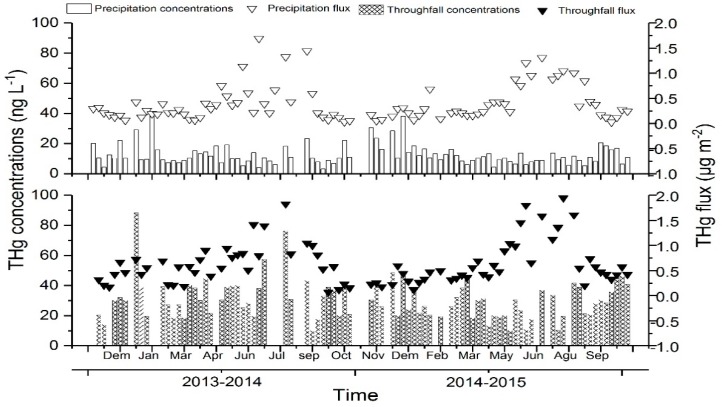
THg concentrations and fluxes in the precipitation and throughfall collected in the study forest stand from 2013 to 2015.

**Figure 3:**
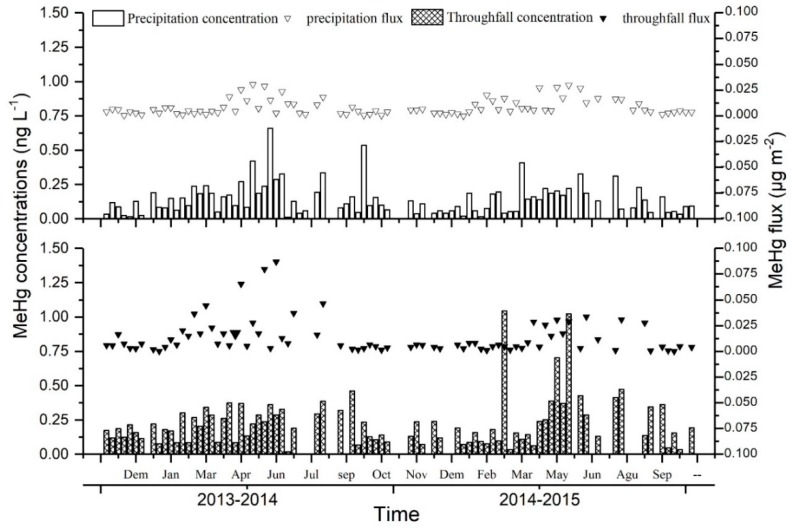
MeHg concentrations and fluxes in the precipitation and throughfall collected in the study forest stand from 2013 to 2015.

Throughfall in the evergreen broadleaf forest possessed significantly higher THg concentrations than the open precipitation (*p* ≤ 0.001, *n* = 104). Mean concentrations of THg in the precipitation ranged from 3.1 to 38 ng L^−1^, and the overall VWM were 10.61 ± 5.6 ng L^−1^ and 9.77 ± 8.3 ng L^−1^ in the first and second year respectively (Table 2). Mean concentrations of THg in the throughfall ranged from 3.2 to 87.3 ng L^−1^ (Figure 2), and the average VWM was 21.28 ng L^−1^ (Table 2). Mean concentrations of MeHg in the direct wet deposition ranged from 0.09 ng L^−1^ to 0.75 ng L^−1^ (Figure 3), and the overall VWM was 0.19 ± 0.13 ng L^−1^ (Table 2). Mean concentrations of MeHg in the throughfall ranged from 0.1 ng L^−1^ to 1.23 ng L^−1^ (Figure 3), averaging 0.24 ± 0.20 ng L^−1^ (Table 2). 

The average fluxes of THg in the litterfall ranged from 47.65 ng L^−1^ to 49.01 ng L^−1^ (Table 2), and the mean fluxes of MeHg in the litterfall collected from the studied forest from 2013 to 2015 ranged from 0.38 ng L^−1^ to 0.39 ng L^−1^ (Table 2).

### 3.2. Dynamics of Hg in the Forest Runoff

**Figure 4:**
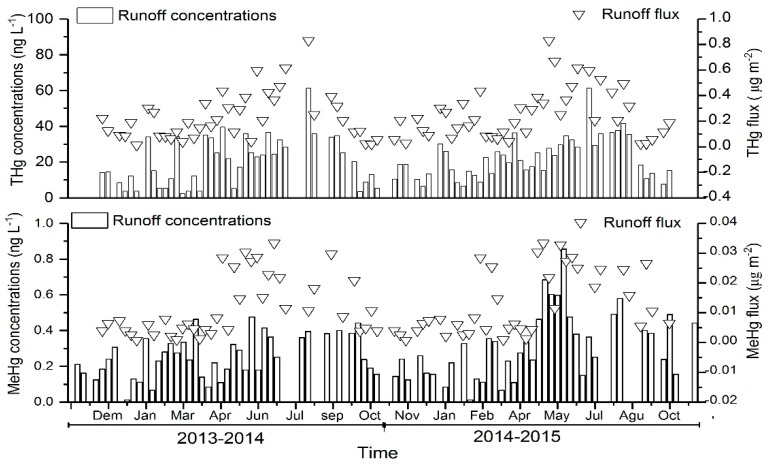
THg and MeHg concentrations and fluxes in the forest runoff collected in the study forest stand from 2013 to 2015.

The variations of unfiltered THg and MeHg concentrations and fluxes in the forest runoff in Mt. Simian were shown in Table 2 and Figure 4. THg concentrations in the forest runoff varied between 11.3 ng L^−1^ and 40.7 ng L^−1^, with the VWM concentration of 21.28 ng L^−1^. The highest concentrations of MeHg in the runoff occurred mainly from May to July (>0.15 ng L^−1^), whereas THg concentrations in the runoff were elevated (>20 ng L^−1^) during the rainy season (June to September) (Figure 4). THg concentrations in the surface runoff were lower than that in the throughfall during the first year, while they were slightly higher than that in the throughfall in the second year. 

### 3.3. Dynamics of Hg in the Forest-Floor Percolates

**Figure 5:**
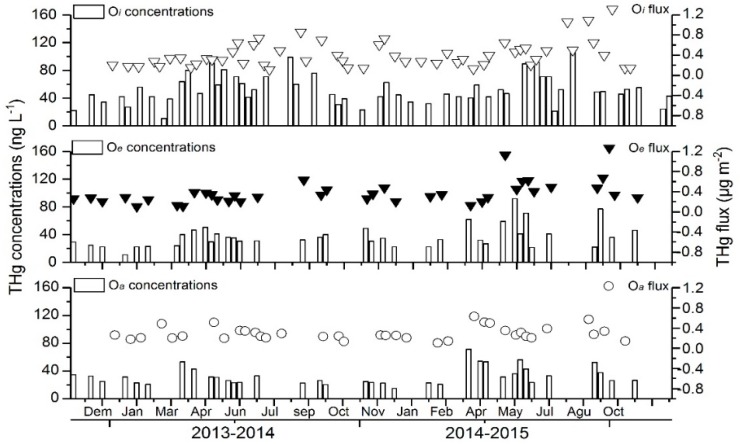
THg concentrations and fluxes in the soil percolates collected in the study forest stand from 2013 to 2015.

**Figure 6:**
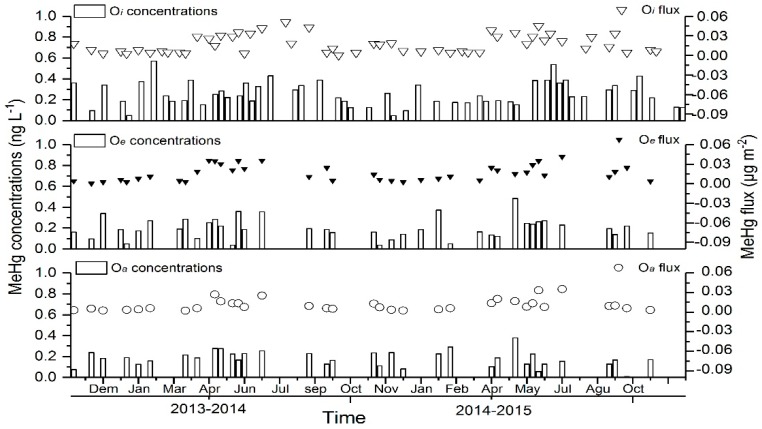
MeHg concentrations and fluxes in the soil percolates collected in the study forest stand from 2013 to 2015.

THg and MeHg fluxes and concentrations were lowest from December to March since it was in the dry season in the studied subtropical forest, characterized by lower temperatures and less rain comparing with other seasons (Figure 5 and Figure 6). The correlation between the throughfall and THg fluxes was highly significant in all the layers (*O_i_*, *O_a_*, and *O_e_*, *p* < 0.001, *t*-test). However, the difference of MeHg fluxes with the throughfall occurred only in the *O_i_* layer, while there was no significant difference in the *O_a_* layer. During the two-year monitoring period, the annual average THg flux in the infiltration water was highest in the *O_e_* layer (11.27), followed by *O_a_* (9.66) and *O_i_* (9.17) layers. One of the sources of THg in the *O_e_* layer could be Hg in the precipitation or throughfall (Table 3).

The concentrations of MeHg was highest in spring, followed by summer and autumn, and lowest in winter. Two-tailed *t*-test results (assuming equal variance) showed that MeHg concentrations in the soil samples in spring had highly significant differences with that in the percolate samples (*p* < 0.001, *t*-test), while the differences between the other seasons were not significant (*p* > 0.05, *t*-test). In autumn, after the plants withered owing to lowered temperatures, the litterfall accumulated MeHg temporarily. The fluxes of MeHg showed significant seasonal variations in soil leachate, particularly in the *O_i_* layer. The fluxes of MeHg in the leachate of *O_i_* layer of the forest soil in spring and summer were significantly higher than that in autumn and winter, and it was lowest in winter. Methylmercury began to accumulate since spring, and it was the highest during late spring and early summer. 

## 4. Discussion

### 4.1. Influence of the Forest Canopy on Hg Deposition through Throughfall

The VWM concentrations of THg and MeHg in the throughfall were found to be 2.0 and 1.3 times higher than those in the precipitation respectively during the experimental period. The concentrations of MeHg and THg in the net throughfall were not found to remarkably correspond with those in wet deposition of the open (*r*^2^ = 0.13), inferring that wet deposition was not the main driver for Hg fluxes in the throughfall, especially for THg. Here net throughfall means throughfall minus direct wet deposition. The variation tendency of MeHg concentrations in the throughfall was somewhat similar to THg. The two-year data indicated that forest canopy had great influence on the concentrations and fluxes of THg. The concentrations of MeHg in the throughfall were also significantly higher than that in the open bulk precipitation (*p* = 0.003, *n* = 107).

THg concentrations in the throughfall were found to be maximum from October to March (cold season), when the rainfall was less and atmospheric Hg concentrations were elevated. The cold season of the sampling site is principally controlled by the northwest monsoon, which generally brings less precipitation. Meanwhile, the cold season of China generally means higher atmospheric stability, which makes atmospheric Hg move difficultly. Therefore, the atmosphere in the cold season possesses higher Hg-scavenging abilities. Contrarily, the sampling site is mainly impacted by southeast monsoon from April to September (warm season), resulting in great increase of rainfall and lower concentrations of Hg (Figure 2).

THg fluxes in the throughfall of Mt. Simian was over 93% enhancement, which was significantly higher than those reported from other places [12,16,18,30]. The reason is perhaps that atmospheric Hg accumulated in the vegetation via dry deposition is washed out by the throughfall. The transportation and deposition of atmospheric Hg is highly dependent on its speciation. It is widely accepted that highly soluble inorganic Hg (Hg(II), Hg^2+^) are rapidly stripped from the atmosphere and deposited locally, whereas aerosol (Hg-p) and elemental (Hg^0^) Hg emissions are transported regionally and globally respectively [30,31,32,33]. Therefore, the enhancement of THg concentration in the throughfall may indicate that the great loading of Hg in the upland forest comes from regional Hg emissions from industrial activities and coal combustion. Canopy density, on the other hand, played certain roles on the elevation of THg and MeHg concentrations. THg concentrations in the throughfall of the evergreen broadleaf forest with canopy densities over 93% were significantly higher than those obtained from other boreal forests of lower canopy densities [32,34]. 

Summarily, THg deposition fluxes at Mt. Simian were significantly higher than those reported from other places in Asia, Europe and North American [10,14,35,36]. Annual Hg flux through thoughfall in Mt. Simian is higher than that in Europe and North American (2.1–40.1 μg m^−2^yr^−1^). To sum up, the total Hg deposition fluxes through litterfall and throughfall in the subtropical forest of Mt. Simian is 2 to 10 times of the observation values in the temperate forests of Europe and North American [9,37,38,39]. The reason perhaps is that forest canopy density of the subtropical evergreen broadleaf forest of Mt. Simian reaches more than 98%, which is exceedingly larger than other sites. More importantly, the increased Hg deposition fluxes over time is probably because of the elevated atmospheric Hg concentrations in the past decades for China’s fast development. This could explain some of the discrepancy from background values observed in European and North American. Meanwhile, both higher THg and MeHg fluxes in the throughfall may lead to the accumulation of Hg in the soils of forest field [30], especially in the warm season (Figure 2).

### 4.2. Influence of the Forest Canopy on Hg Deposition through Litterfall

The average fluxes of THg and MeHg in the litterfall were considerably higher than those found in the forest ecosystems of Norway, Canada and the USA [13,14,16,30,40,41,42] and the global natural background value (0.7–1.1 ng m^−2^ h^−1^) [7]. Our results unequivocally illustrated that the forest canopy provided significant additional fluxes of both MeHg and THg to the forest ecosystems mainly through litterfall. As a matter of fact, the total fluxes of MeHg and THg under a standard 25-year old evergreen forest canopy at Mt. Simian is 2.2 and 2.4 times of those in the open. As we discussed above, the concentrations or fluxes of Hg in the litterfall appear to have correlation with the average atmospheric Hg levels in the local atmosphere. Numerous studies have demonstrated that elevated atmospheric Hg levels could promote the stomatal and nonstomatal uptake of Hg by the foliage [13,35,36]. As mentioned above that soluble Hg were easy to strip from the atmosphere and deposit locally, while aerosol emissions were transported regionally [14]. This result implies that a large majority of aerosol or reactive gaseous Hg were accumulated on the foliage via dry deposition in the study site. Therefore, our results further indicated that more serious atmospheric Hg pollution might contribute to greater fluxes to soils through throughfall and litterfall in the forest land, which was the same with previous results [38].

### 4.3. Dynamics of Hg in the Forest Runoff

THg concentrations in the surface runoff were generally higher in summer and autumn, but lower in winter. The underlying reason could possibly be that organisms (especially the flora and fauna) could decompose the organic matter in the litter and soil. Moreover, the mobility of released THg in the soil solution is affected by the soil moisture which is lower in the autumn and winter. Mercury as a heavy metal could also be directly adsorbed by the litter and soil, thereby reducing its activity [6,43,44,45]. Therefore, the exogenous input of Hg in the soil and litter would undergo a process of gradual accumulation. Mercury adsorbed and intercepted by leaves would eventually return to the forest soil owing to microbial decomposition of litter. However, the leaching of forest soil litter and organic matter causes great desorption effects on Hg in the soil. This might because that the solution in the organic matter contains a large amount of dissolved organic material (DOM). Previous studies have shown that since soil DOM contained a large number of functional groups, and they could form organometallic complexes with Hg by chelation, thus elevating the solubility of Hg [31,36]. In the litter and soil, the leaching of DOM towards heavy metals was particularly pronounced [46]. In addition, the increased amounts of Hg and active Hg in the newly settled litter could be fixed by a series of microbial activities during the decomposition process. This is particularly evident in the warm and humid summer, resulting in the accumulation of Hg in decayed leaf litter and soil humus layers, which could be washed away after the rain. The fluxes and concentrations of Hg in the surface runoff were temporally constant, unlike that in the precipitation and throughfall [47]. During heavy rainfall events, the precipitation and throughfall will dilute Hg in their respective environments, causing low Hg concentrations in the precipitation and throughfall. However, heavy rainfall resulted in high Hg fluxes. Furthermore, according to the characteristics of the surface runoff generation, the stronger the precipitation intensity, the greater the surface erosion. This phenomenon probably causes soluble Hg in the surface runoff to be larger, thereby increasing Hg fluxes [6,43,44,45].

MeHg concentrations in the surface runoff were significantly higher than that in the precipitation and throughfall. During the study period, higher values were recorded from April to June, which might be due to the warm and humid climate which favors microbial activities in the litter and soil, particularly those reductive anaerobic microorganisms. Mercury accumulated in decaying leaf litter could be absorbed or fixed by the litter with the help of microorganisms. Subsequently, this part of Hg could be transported into the soil in the form of residues and be covered by the subsequent batches of litter, forming a good anaerobic environment [13,48,49]. Coupled with warm and humid forest soil conditions, this could facilitate the production of MeHg in the decomposed litter, which migrates during the erosion by surface runoff. Consequently, MeHg concentrations were quite consistent with that in the surface runoff.

### 4.4. Dynamics of Hg in the Forest-Floor Percolates

Results indicated that the correlation between the throughfall and THg fluxes was highly significant in all the layers. It is known that water on the soil surface increased or reached saturation after raining, thereby causing water soluble Hg in the soil to move down gradually along with the soil solution into the *O_e_* layer. The permeability coefficients of *O_i_* and *O_e_* layers in the forest soils of Mt. Simian were 0.00602 cm·s^−1^ and 0.00153 cm·s^−1^ respectively. Obviously, the former was approximately four times of the latter. According to the characteristics of subsurface runoff generation, when the precipitation and throughfall intensities are both large, the forest soil percolate will produce two different forms of flows. One is at the interface between the *O_i_* layer and *O_e_* layers, and the other is at the interface between *O_a_* layer and the rock layer. The former flows fast and the latter is slow. With increasing flow, the former shows a trend of flowing slower than the latter. Therefore, *O_e_* layer had higher soluble Hg concentrations. Mercury in the throughfall and accumulated in the litter could have been absorbed by decomposed litter or fixed on the surface of leaf litter by microbial activities. Eventually, this part of Hg would enter into the forest soil in the form of residues and be covered by the subsequent batches of litter. However, if Hg in the lower soil layer and more readily decomposable litter migrated with the new litter and the fungal colony, this part of Hg would constitute the internal circulation mechanism of the forest, whereby the Hg will accumulate at the top layer of the soil. Thus, Hg transfer through the cross-section of the soil would be delayed, thereby increasing the retention time of Hg in the forest soil. As the leaves of trees and their litter eventually decompose and integrate with the soil, the *O_e_* and *O_a_* layers of the soil might store and enrich Hg in the forest ecosystem. 

The concentrations of MeHg were highest in spring, followed by summer and autumn, and lowest in winter. It seemed that MeHg began to accumulate since spring, and it reached maximum in late spring and early summer. The reason could be that the decomposition rate of organic matter in newly settled litter was slow, and input of Hg through the litter was greater in autumn. However, the large amount of Hg input could probably convert to MeHg in the warm and humid spring and summer by a series of fixation and conversion by microorganisms [14]. As mentioned above that Hg in the throughfall and litter could be absorbed or fixed by the litter by microbial activities. Therefore, this part of Hg would eventually transform to the underlying soil in the form of residues, and then be covered by the subsequent fluttered litter. However, if Hg accumulated in the more readily decomposable litter and upper soil layer were fixed by microorganisms below the newly fallow litter, this part of Hg would circulate internally in the forest. Since Hg is accumulated in the lower layer of the litter and upper layer of the soil, its transfer through the soil cross-section is delayed, contributing to elevated retention time for MeHg in the forest soil that was easily eroded by the rainfall.

## 5. Conclusions

The forest field exerts essential roles in the biogeochemical cycling of THg and MeHg in the forest ecosystems. It can be regarded as an effective converge for MeHg in the upper forest field, since only a small part of the deposited MeHg migrates into the lower soil layers. In contrast, THg retention is not consistent with MeHg. This particular phenomenon indicates that the recovery of the forest floor from historical THg accumulation is delayed by an enhanced permeability and retention effect of the adsorption of THg. Although there is different transferability of the two compounds in the forest floor and varied influencing factors influences their release between the runoff and percolate, these differences do not have a concurrent variation in the input-output budget of the entire ecological system at the interannual scale. However, it means that, for other factors of MeHg in the ecological system (like methylation in the litterfall), further research needs to be done to discover a more precise forecast model. Furthermore, it is not certain whether human Hg exposure exhibits via the runoff or interflow with the increasing accumulation of Hg in soil. The deposited Hg, especially the MeHg, gathered in the decomposing litter and upper soil, and the transfer of these two compounds by soil percolate has been delayed. This could increase the residence time of MeHg in the forest land once non-renewable harvesting of biomass contributes to deforestation and thus produces an ecological risk. In addition, there is not a systematic and precise study to explain the migration and transformation of Hg in forest watersheds due to the complexity of Hg geochemical behavior and the limitations of observation and experimental methods, as well as the imperfect parameterization schemes for models. Therefore, future work on establishing new technologies and methods for quantifying the storage, migration, and transformation of Hg in forest systems should be conducted in order to obtain more exact results.

## Figures and Tables

**Table 1:** THg and MeHg concentrations in different layers of the forest floor.

Layer		THg (ng·g^−1^)	MeHg (ng·g^−1^)	Density (g cm^−3^)	Thickness (m)	THg Content (μg·m^−2^)	MeHg Content (μg·m^−2^)
Litterfall		73.3 ± 14.2	0.86 ± 0.4	0.38 ± 6.2	0.21	5849.3	8.9
Soil	*O_i_*	315.5 ± 27.3	1.06 ± 0.3	1.37 ± 2.1	0.20	9509.1	31.9
	*O_e_*	408.6 ± 42.2	0.61 ± 0.2	1.54 ± 5.9	0.20	20,106.2	30.1
	*O_a_*	368.4 ± 22.7	0.26 ± 0.2	1.57 ± 16.6	0.20	25,449.1	17.9

*O_i_* means the upper soil with depth of 0–20 cm; *O_e_* indicates soil with depth of 20–40 cm, while *O_a_* means soils at the depth of 40–60 cm.

**Table 2:** The corrected concentrations and fluxes of THg and MeHg in the throughfall, litterfall, runoff, forest-floor percolates and water during the sampling period from 2013 to 2015.

Samples	THg Concentration		MeHg Concentration		Water/Litterfall Flux		THg Flux		Mehg Flux	
	**ng L^−1^**				**mm**		**μg m^−2^ yr^−1^**			
	**2013–2014**	**2014–2015**	**2013–2014**	**2014–2015**	**2013–2014**	**2014–2015**	**2013–2014**	**2014–2015**	**2013–2014**	**2014–2015**
Precipitation	10.61 ± 5.6	9.77 ± 8.3	0.13 ± 0.12	0.11 ± 0.13	1395	1480	16.19	18.89	0.18	0.16
Throughfall	22.14 ± 4.9	20.92 ± 16.2	0.20 ± 0. 08	0.19 ± 0.22	1073	1188	22.34	24.35	0.23	0.22
Runoff	20.32 ± 9.1	22.24 ± 19.4	0.31 ± 0.19	0.29 ± 0.18	861	884	19.93	21.66	0.27	0.26
Percolate (*O_i_*)	45.59 ± 12.1	48.29 ± 34.2	0.26 ± 0.12	0.25 ± 0.16	582 (834)	602 (850)	8.03	10.20	0.22	0.21
Percolate (*O_e_*)	55.84 ± 10.2	56.58 ± 28.3	0.21 ± 0.16	0.22 ± 0.14	584 (896)	610 (867)	10.54	11.95	0.15	0.16
Percolate (*O_a_*)	51.05 ± 25.5	55.14 ± 19.6	0.19 ± 0.11	0.17 ± 0.08	591 (808)	604 (829)	9.27	10.04	0.15	0.14
Litterfall	**ng g^−1^**				**g m^−2^**		**μg m^−2^ yr^−1^**			
	104.5 ± 23.5	106.3 ± 18.7	0.84 ± 0.12	0.83 ± 0.20	456	461	47.65	49.01	0.38	0.39

**Table 3:** The corrected concentrations and fluxes of THg and MeHg in the runoff, forest floor percolates and water during the sampling period from 2013 to 2015.

Layer	THg Concentration (ng L^−1^)		MeHg Concentration (ng L^−1^)		Water/Litterfall Flux (mm)		THg Flux (μg m^−2^ yr^−1^)		MeHg Flux (μg m^−2^ yr^−1^)	
	2013–2014	2014–2015	2013–2014	2014–2015	2013–2014	2014–2015	2013–2014	2014–2015	2013–2014	2014–2015
Runoff	20.32 ± 9.1	22.24 ± 19.4	0.31 ± 0.19	0.29 ± 0.18	861	884	19.93	21.66	0.27	0.26
